# Choline Improves Neonatal Hypoxia-Ischemia Induced Changes in Male but Not Female Rats

**DOI:** 10.3390/ijms232213983

**Published:** 2022-11-12

**Authors:** Tayo Adeyemo, Ayodele Jaiyesimi, Jill G. Bumgardner, Charity Lohr, Aditi Banerjee, Mary C. McKenna, Jaylyn Waddell

**Affiliations:** 1 Department of Pediatrics, University of Maryland School of Medicine, 655 W. Baltimore St., Baltimore, MD 21201, USA; 2 Program in Neuroscience, University of Maryland School of Medicine, Baltimore, MD 21201, USA

**Keywords:** hypoxia ischemia, neonate, choline, sex differences, eyeblink conditioning, neurite outgrowth

## Abstract

Choline is an essential nutrient with many roles in brain development and function. Supplementation of choline in early development can have long-lasting benefits. Our experiments aimed to determine the efficacy of choline supplementation in a postnatal day (PND) 10 rat model of neonatal hypoxia ischemia (HI) at term using both male and female rat pups. Choline (100 mg/kg) or saline administration was initiated the day after birth and given daily for 10 or 14 consecutive days. We determined choline’s effects on neurite outgrowth of sex-specific cultured cerebellar granule cells after HI with and without choline. The magnitude of tissue loss in the cerebrum was determined at 72 h after HI and in adult rats. The efficacy of choline supplementation in improving motor ability and learning, tested using eyeblink conditioning, were assessed in young adult male and female rats. Overall, we find that choline improves neurite outgrowth, short-term histological measures and learning ability in males. Surprisingly, choline did not benefit females, and appears to exacerbate HI-induced changes.

## 1. Introduction

Hypoxic ischemic encephalopathy (HIE) is defined as abnormal neonatal brain function resulting from an acute perinatal hypoxic/ischemic event [1,2]. The incidence in developed countries is 1.5/1000 live births and it is a major cause of mortality and neurologic morbidity [3]. Up to 30% of infants have impairments that can range from cerebral palsy to visual and hearing impairments and 15–20% of affected neonates die shortly after birth [1,2]. Currently the standard of care for treatment of moderate to severe HIE in infants greater than 36 weeks gestation is therapeutic hypothermia, which improves survival and neurologic outcomes and reduces further brain damage [4,5]. Despite these benefits, the neuroprotection hypothermia affords is not complete [6,7]. Many studies have explored adjunct therapies to further improve neuroprotection after hypoxia ischemia (HI) injury, including nutritional supplementation, but to date, success has been limited [3,4].

Choline is an essential nutrient with a variety of roles including regulation of gene expression, neurotransmission, cellular membrane integrity, and methionine synthesis [8,9,10]. Choline plays a key role in 1- carbon metabolism and is the precursor for important compounds including acetylcholine, phospholipids, and betaine, a key methyl group donor in metabolism [10]. Early studies of choline supplementation in rats found that perinatal administration improved cognition in adulthood, an effect termed “metabolic imprinting” [11,12,13]. Importantly, these effects were observed in typically developing rats, without choline deprivation. Choline can minimize symptoms of Trisomy 21, Rett syndrome, Alzheimer’s disease and the effects of maternal infection in children when given prenatally [14,15,16,17,18]. Choline supplementation has also reduced learning deficits in rodent models of fetal alcohol spectrum disorder and in children exposed to alcohol prenatally [19,20,21,22,23]. Deficiency of choline can lead to increased mutations, deleterious changes in metabolism of other critical nutrients such as folate and permit excessive apoptosis in the developing brain [8,9,10]. A recent kinetic study of choline in 14 day old rat pups found that choline metabolites accumulated in brain between 6–24 h after administration of D9-choline, leading to pools of phosphocholine and CDP-choline that were higher in the cerebrum and cerebellum than in liver [24]. The authors noted that the high proportion of labeled choline metabolites in brain, including phosphatidyl choline and sphingomyelin, was evidence of the high demand for choline in brain development [24].

Despite the breadth of research regarding choline’s benefits for brain development, few studies have tested the efficacy of choline administration in neonatal HI. Oral administration of choline can increase levels of choline and its metabolites in the brain, making supplementation relatively simple [8]. The cholinergic system is vulnerable to HI [25]. The number of acetylcholinetransferase positive neurons was reduced in the septum and morphological dysregulation of this population of neurons was observed in the nucleus basalis of Meynert after HI [25]. Many in vivo magnetic resonance spectroscopy (MRS) studies have detected changes in choline and its metabolites after HIE, suggesting disrupted structural integrity and cell membrane turnover [8,26,27]. Brain choline concentrations are reduced in infants suffering severe injury [27,28,29]. Administration of citicoline, an intermediate in the synthesis of phosphotidylcholine from choline, improved crude histological measures of brain volume in a rat HI model, and also reduced caspase 3 expression [30]. A very recent study demonstrated that a high nutrient diet, including omega-3 fatty acids, uridine monophosphate, zinc, vitamin B12 and choline reduced tissue loss and neuroinflammation in males rat pups subjected to HI [31]. Interestingly, no benefit was observed in females [31]. 

Our experimental goal was to determine whether postnatal choline supplementation is neuroprotective in male and female rat pups subjected to HI using the Rice-Vannucci model of injury in a term infant [32,33]. We utilized a sex-specific in vitro neurite outgrowth assay to determine the effects of choline supplementation on cerebellar granule neurons in sex-specific culture from pups subjected to HI on P10 or sham controls with and without choline administration. The effects of choline on both motor behavior and learning ability were tested in young adulthood. Histological quantification of cerebral injury was conducted on tissue collected 72 h after HI and in adult rats tested in the behavioral experiments. Overall, choline does improve neurite outgrowth in male-derived cells, and improves learning ability in adulthood. Motor performance was not significantly affected by HI in either sex, as results were variable. Surprisingly, choline did not improve outcomes in females, and tended to exacerbate HI-induced changes.

## 2. Results

### 2.1. Neurite Outgrowth in Sex-Specific Cultured Cerebellar Granule Cells after HI

**Figure 1:**
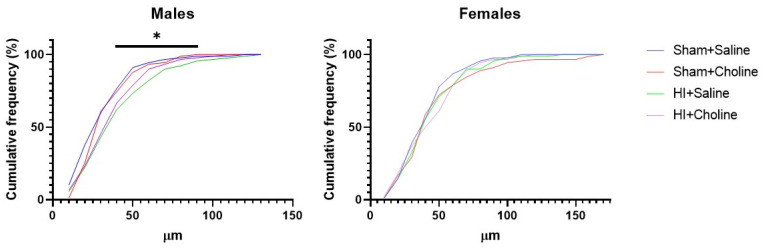
Choline supplementation restores neurite outgrowth in cerebellar granule cells derived from male pups subjected to HI. Cumulative frequency distributions of neurite lengths of cultured cerebellar granule cells. Neurite lengths were measured in neurons harvested from pups subjected to HI on PND10. Cells were fixed and stained for analysis 48 h after plating. Thirty cells per animal were quantified, from 3 animals per group for a total of 90 cells per group. Data are presented in 10 μm bins. Kruskall Wallace ANOVA found a significant effect of group for male-derived cells, * = *p* < 0.008. HI reduced neurite length in male-derived cells only. Neurite lengths were shorter in the HI+saline group compared to the Sham + saline group, KS test, *p* < 0.009. Choline partially reversed this effect as HI + Choline derived cells did not differ from any other group, *p* > 0.05. No differences were detected in female-derived cells. HI = hypoxia ischemia.

**Figure 2:**
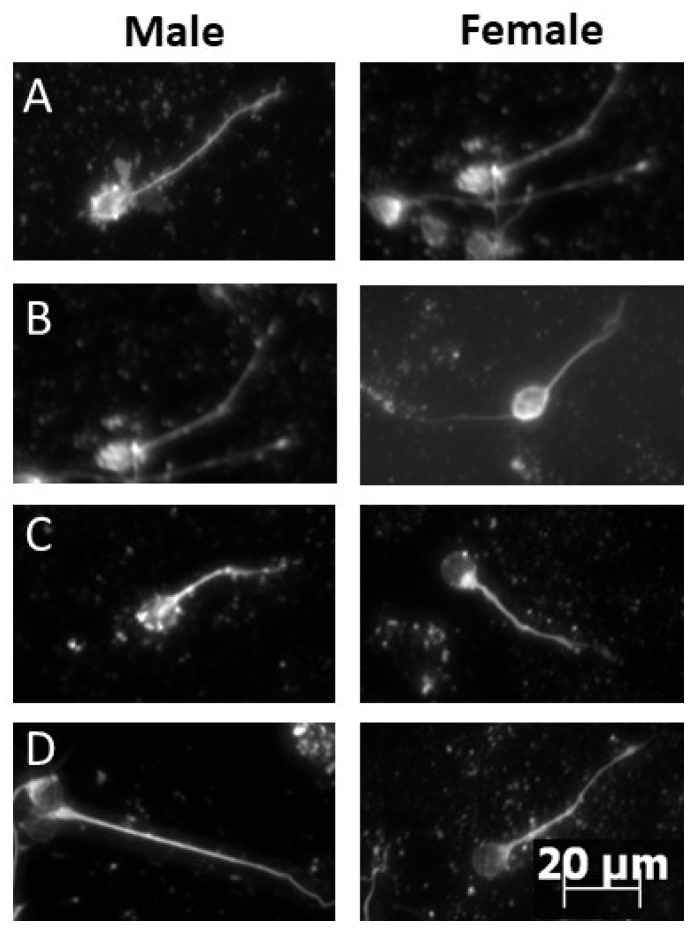
Hypoxia-ischemia reduced neurite outgrowth in male-derived cerebellar granule cells and this was improved by choline. Representative images of cultured cerebellar granule cells measured in neurons harvested from pups subjected to HI on PND10 (data presented in Figure 1). Representative cells from (**A**): Sham + saline males (**left**) and females (**right**); (**B**): Sham + choline males (**left**) and females (**right**); (**C**): HI+saline males (**left**) and females (**right**); (**D**): HI + choline males (**left**) and females (**right**). Cells were fixed and stained for analysis 48 h after plating.

We analyzed neurite length of cultured cerebellar granule cells collected 4 h after HI on P10. Male and female pups treated with either choline or saline and subjected to either HI or sham control surgery were individually cultured for 48 h prior to immunocytochemistry. Kruskall Wallace ANOVA determined a significant group difference in cells derived from male cerebellar granule cells, *H*(4) = 11.68, *p* < 0.008. Male sham saline compared with HI saline using a KS test found a significant difference, KS D = 0.244, *p* < 0.009 (Figure 1), confirming that HI shifted the distribution of neurite length compared to sham operated controls. Neurite lengths did not differ between HI with saline or HI with choline administration, *p* > 0.05 and sham-derived cells did not differ from cells derived from HI pups treated with choline, *p* > 0.05. This pattern of results suggests that while choline improved neurite outgrowth, it did not completely restore growth. No significant differences were detected in cells derived from female pups, (Figure 1). Representative images of cultured cerebellar granule cells from each experimental group are presented in Figure 2. 

### 2.2. Magnitude of Injury as Determined by Area of Ipsilateral and Contralateral Hemispheres

**Figure 3:**
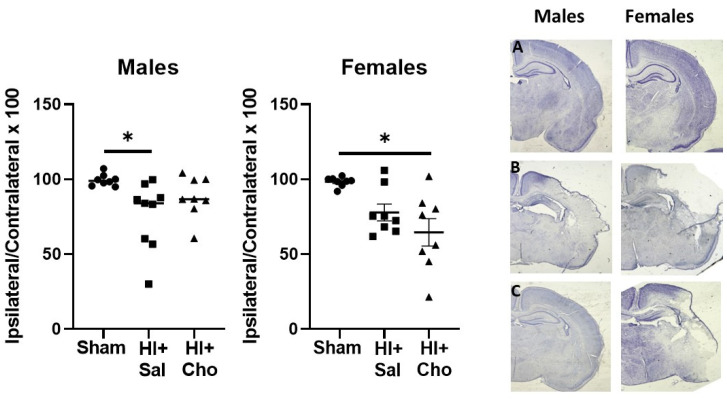
Choline supplementation improved histological outcomes at 72 h after HI in males but not females. Cerebral hemisphere volume was measure in sham operated control pups and pups subjected to HI on PND10. Tissue was collected 72 h after HI. The area of the contralateral and ipsilateral hemispheres was measured in 3 sections from a 1:12 series containing the dorsal hippocampus. Data are expressed as area of the ipsilateral/area of contralateral × 100. Male pups subjected to HI exhibited significant tissue loss compared to sham controls; only females treated with choline were significantly different from sham controls, * *p* < 0.05. Each group, n = 8. Representative images of the ipsilateral hemisphere of (**A**): Sham male (**left**) and female (**right**) pups; (**B**): HI+Sal male (**left**) and female (**right**) pups; (**C**): HI+Cho male (**left**) and female (**right**) pups. HI = hypoxia ischemia; Sal = Saline; Cho = Choline.

The volume of the cerebral hemisphere was smaller in HI + saline males compared to sham males. Analysis of males found a significant effect of group, *H*(3) = 7.14, *p* < 0.02, Figure 3. Sham and HI + saline males were significantly different as determined by Dunn’s multiple comparison test, *p* < 0.01. Sham males did not differ from HI + choline (*p* > 0.05) and the HI groups did not differ from each other, *p* > 0.05. Analysis of females also confirmed a significant effect of group, *H*(3) = 8.64, *p* < 0.01. Comparison of sham females and HI + saline pups found a marginal difference between sham operated and saline treated HI females, *p* < 0.056. Sham operated females were significantly different than HI females treated with choline, *p* < 0.01. The HI groups (i.e., HI + Saline vs. HI + Choline) did not differ from each other, *p* > 0.05. This pattern of results suggests that choline improved histological outcomes in males, but not females.

### 2.3. Behavioral Outcomes following HI with or without Choline Administration

#### 2.3.1. Dowel Crossing and Rotarod Tests 

**Figure 4:**
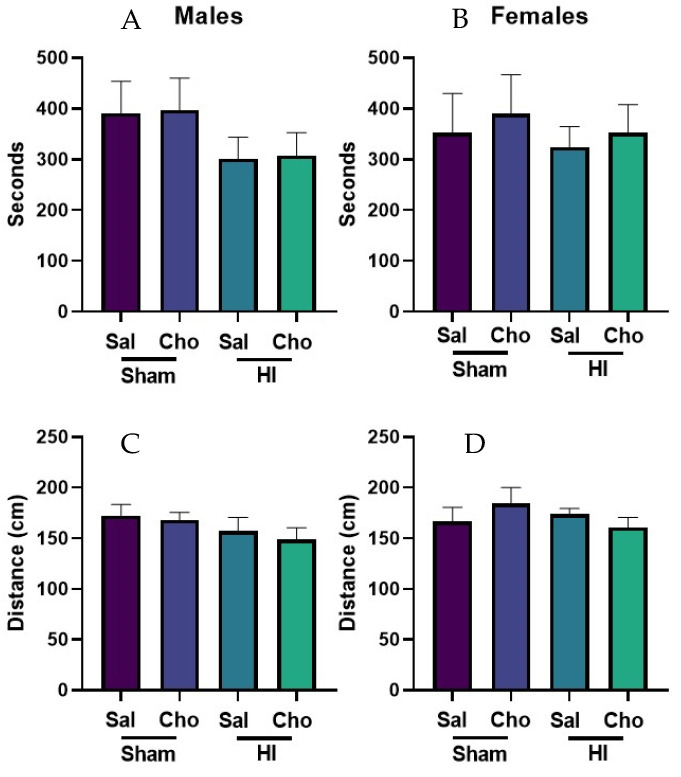
Motor performance was not significantly changed by hypoxia ischemia or choline in either sex. Neither HI nor choline administration significantly affected rotarod performance in males (**A**) or females (**B**). The total time spent on an accelerating rotarod was calculated. Data were collected across 3 days, with 3 trials per day. Dowel crossing performance was not significantly affected by HI or choline administration in male (**C**) or female (**D**) rats. The total distance traversed over 3 days consisting of 3 training trials each was calculated for analysis. Sham groups consisted of 6–7 rats per group; HI groups consisted of 8–11 rats per group. Sal = Saline; Cho = Choline; HI = hypoxia ischemia.

We tested rats in two common motor tasks, dowel crossing and the accelerating rotarod, between PND30-39. ANOVA did not find a significant effect of HI or choline on either the dowel crossing test or rotarod performance, *p* > 0.05 (Figure 4). Thus, motor performance was unchanged by HI, and there was no benefit of choline administration in either HI or sham control animals.

#### 2.3.2. Eyeblink Conditioning 

**Figure 5:**
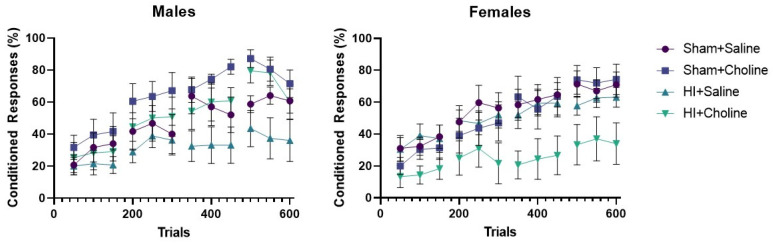
Choline improved cerebellar-dependent learning in males subjected to hypoxia ischemia but not females. Male and female rats were tested in a trace eyeblink Pavlovian conditioning task in young adulthood. Data are presented in 50-trial blocks. Rats were presented with 150 training trials per day. Left Panel: Choline administration improved performance of male rats regardless of the surgery condition, *p* < 0.02. HI males treated with saline exhibited poor learning across days, and this was significant for the last two days of training, *p* < 0.03. Right Panel: Choline treated females performed poorly across all four training days. Analysis of the last two training days indicated a significant surgery × treatment interaction, because only HI females treated with choline performed poorly, *p* < 0.04. Choline did not affect behavior in sham control females and HI females treated with saline outperformed females treated with choline. Sham control groups consisted of 6–7 rats each; HI groups consisted of 7–9 rats each. HI = hypoxia ischemia.

The trace eyeblink task is a Pavlovian conditioning preparation that can test cerebellar, hippocampal and prefrontal cortex function [34]. In young adulthood (PND50-54), rats from sham and HI groups with and without choline were trained with a tone paired with a periorbital stimulus separated by a brief gap, referred to as the ‘trace’. Males and females were analyzed separately. Repeated measures ANOVA of performance in males confirmed a significant effect of trial, as the percentage of conditioned responses expressed increased across trial blocks in most groups, F(11,286) = 22.45, *p* < 0.001 (Figure 5, left panel). The trial × choline treatment interaction was significant, F(11,286) = 2.39, *p* < 0.007. No other interactions were significant. The between subjects main effect of surgery did not quite reach significance, F(1,26) = 3.17, *p* < 0.07 when trial blocks from all 4 training days were analyzed, due to the fact that choline improved performance in the male HI group. The main effect of choline treatment was significant, F(1,26) = 5.77, *p* < 0.02. The surgery × choline treatment interaction was not significant. These results confirm that choline improved performance of males regardless of the surgery condition. Repeated measures ANOVA of the last 2 days of training found a significant effect of surgery, F(1,26) = 4.76, *p* < 0.03 and choline, F(1,26) = 8.27, *p* < 0.008. HI males treated with saline exhibited fewer conditioned responses than sham operated or choline treated males. 

Repeated measures ANOVA of females’ performance (Figure 5, right panel) also confirmed a significant effect of trial, F(11,275) = 2.52, *p* < 0.001. This confirms that the number of conditioned responses increased as training trials progressed in most groups. The trial × surgery interaction was significant, F(11,275) = 2.75, *p* < 0.001. No other interactions were significant. The main effect of surgery was significant, F(1,25) = 5.13, *p* < 0.03. This is driven by the poor performance observed in HI females treated with choline. The main effect of choline was significant, as the HI + choline group performed poorly compared to all other groups F(1,25) = 4.80, *p* < 0.03. Isolation of the last two days of training confirmed a significant effect of surgery, F(1,25) = 8.73, *p* < 0.007 as well as a surgery × choline interaction, F(1,25) = 4.42, *p* < 0.04. Only HI females treated with choline were still exhibiting poor performance in the latter half of training. 

**Figure 6:**
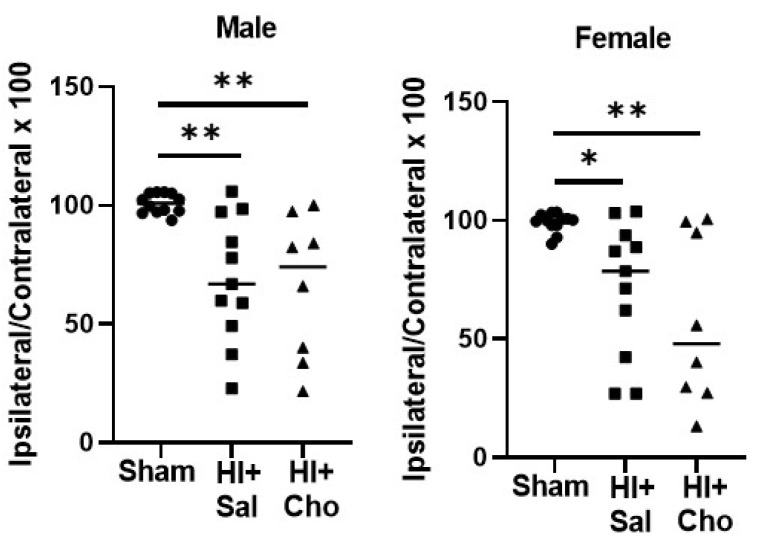
Choline did not significantly improve long-term histological outcomes in males or females. Cerebral hemisphere volume was measured in young adult rats tested behaviorally after HI on PND10. The area of the contralateral and ipsilateral hemispheres was measured in 3 sections through the dorsal hippocampus. In both males and females, HI groups differed from sham controls regardless of saline or choline treatment, * *p* < 0.05, ** *p* < 0.01 Data are expressed as area of the ipsilateral/area of contralateral × 100. HI = hypoxia ischemia; Sal = Saline; Cho = Choline.

We analyzed cerebral volume from rats behaviorally tested to determine whether the effects of choline were detected in adulthood (Figure 6). Kruskall Wallace ANOVA found a significant effect of group in males, *H*(3) = 12.25, *p* < 0.002. We did not observe a difference between HI males in the saline versus the choline condition at this timepoint, *p* > 0.05, and both HI saline (*p* < 0.006) group and HI choline (*p* < 0.005) group did differ from sham operated controls. This was true for female rats as well. Kruskall Wallace ANOVA confirmed a significant effect of group, *H*(3) = 9.76, *p* < 0.007. There was no significant difference between HI rats treated with saline or choline, and both HI saline (*p* < 0.02) and HI choline (*p* < 0.01) groups significantly differed from sham operated controls.

## 3. Discussion

Our results confirm a beneficial effect of choline during the perinatal phase in male rat pups subjected to HI at term equivalent (PND10). Administration of choline from PND1-10 partially normalized neurite outgrowth in cerebellar granule cells derived from male HI pups and reduced tissue loss in the ipsilateral cerebral hemisphere when measured 72 h after HI. However, this effect did not persist into adulthood as no significant histological difference between male HI pups treated with choline compared to those treated with saline was detected. Acquisition of the conditioned eyeblink response was also improved by choline administration in males. These effects were not observed in females. Female derived cerebellar granule cells did not exhibit a change in neurite outgrowth after HI. Importantly, we did not observe a statistically significant difference between female sham controls and saline-treated females subjected to HI when tissue loss was assessed 72 h after HI. However, female pups treated with choline after HI appear to be more severely affected in both the cerebral volume measures and eyeblink conditioning. These unexpected results demonstrate a novel sexual dimorphism in response to choline in our model of HI. Importantly, choline did not affect motor behavior in either sex, and did not impair eyeblink conditioning in sham operated females. Together, this suggests that choline supplementation exacerbated HI injury in female rat pups, and impacted histology and learning related plasticity negatively only in this group. 

Developmental choline supplementation promotes efficient cognitive function throughout the lifespan in healthy rats [11,12]. The majority of the seminal studies in rodents, however, were conducted only in male rats [11,12,13]. We have observed beneficial effects of choline in females and males exposed to prenatal ethanol on measures of working memory and executive function using the same dose of choline used here, suggesting that the sex difference we observe does not necessarily generalize to healthy rats, or other models of perinatal insult [21,35]. Choline supplementation during pregnancy has been shown to improve visual memory, processing speed and facilitate language and motor development when tested in early childhood, and this does not appear to be dependent upon the sex of the infant [36,37,38]. Moderate to high maternal serum choline levels during pregnancy protect the fetus from the effects of maternal infection, as determined by measures of processing speed and self-regulation in early childhood [17,18]. While the beneficial effects are not absolutely sex dependent, developmental sex differences in choline metabolites are known. In healthy human infants, females exhibit higher levels of choline metabolites in the thalamus, superior frontal gyrus and premotor region than males [39]. Within the same infants, females also had higher levels of *N*-acetylaspartate (NAA) and creatine [39]. Rapidly increasing postnatal levels of NAA predict positive cognitive outcomes, as NAA is considered a marker of neuronal mitochondrial function and maturation [39,40]. The sex differences in metabolites reported in human infants suggest that brain maturation is faster in females than males at this stage, since these metabolites increase quickly in early development [41,42]. Thus, further supplementation might not be as effective in females compared to males or females may not require as much choline supplementation as males. It is noted that in many studies of HI and other perinatal insults such as gestational ethanol exposure, females do not exhibit as severe of neurological changes or behavioral deficits as males, making assessment of neuroprotection in both sexes more difficult [21,35,43,44]. Our results do parallel those reported by Brandt et al. in which a high nutrient diet only benefitted males and not females subjected to HI [31]. Thus, nutrition-based interventions in HI may produce more consistent divergence between the sexes than previously recognized.

The conditioned eyeblink reflex is a sensitive measure of learning-dependent plasticity. In human infants, the ability to acquire a well-timed eyeblink predicts social behavior and a variety of neurodevelopmental disorders that might not be diagnosed until much later in life [45]. The parameters of the trace eyeblink conditioning procedure used here require communication between the prefrontal cortices, hippocampus and cerebellum [46,47]. The observation that choline improved eyeblink performance in male pups suggests that communication between these brain regions was improved, or plasticity within these regions was improved by this therapy. Choline improves eyeblink conditioning in both rodents and humans exposed to prenatal ethanol [48,49,50]. In a study conducted only in male rats the effects of choline were more robust when eyeblink conditioning required cooperation between these brain regions than when the task relied only upon the cerebellum [50]. Sex differences in eyeblink conditioning are well established, as well as divergence between the neural circuits that modulate acquisition of this reflex [51,52,53]. The present study, to our knowledge, is the first to find a sex difference in the response to choline after injury using this sensitive behavioral measure. Our study and the results of others [31] highlight the need for further research with a focus on the female HI model and the possible need for sex-specific therapies to yield improved outcomes in HI. 

An important limitation of our study is the use of only a single dose and timing of choline administration. Dams were maintained on a standard lab chow diet that was not explicitly choline deficient. Therefore, we cannot conclude that choline is necessarily toxic in females in our model of HI. It is possible that this dose is too high, particularly if sufficient levels of choline are available through diet. Another limitation is that we directly administered choline to pups postnatally. The first two weeks of postnatal development in the rat is akin to the last trimester of gestation in humans but does not model the nutritional interaction between the mother and the fetus [54]. Recent work in humans suggests that prenatal choline supplements change biomarkers of docosahexaenoic acid metabolism in cord blood at the time of delivery, highlighting complex interactions between nutrients such as DHA and the importance of dietary methyl-donors during pregnancy [55]. Our model does not capture this complexity. Nevertheless, we observe a striking sex difference in the response to choline in pups subjected to a well-accepted model of neonatal HI. 

## 4. Methods

### 4.1. Animals

All procedures were conducted in compliance with the Institutional Animal Care and Use Committee (IACUC) of the University of Maryland School of Medicine. Our studies used timed pregnant female Sprague Dawley rats acquired from Charles River. Dams were permitted to deliver normally in the animal care facility. The day of birth was considered postnatal day 1 (PND1). Each litter of pups was culled to a maximum of 12 pups, with equal males and females as best possible.

### 4.2. Choline Administration

The day after birth, pups were randomly assigned to either choline or saline experimental condition. Choline was dissolved in sterile saline and administered via a subcutaneous injection at a dose of 100 mg/kg. Each pup received either choline or an equivalent volume of saline on PND1-10 prior to undergoing the hypoxic ischemia procedure for both cell culture/neurite outgrowth and histology experiments. Pups reared for behavioral tasks were treated on PND1-14 and perfused PND57-59. 

### 4.3. Hypoxia Ischemia Procedure

To model the term infant, a modified Rice-Vannucci procedure was conducted on PND10 in male and female pups. The dam and her pups were transported to the laboratory and each pup’s weight and sex was recorded. The pups were then randomly assigned to an experimental condition. Pups assigned to the HI group were anesthetized with isoflurane and the right carotid artery was ligated twice and severed between the ligations. The pup received an external suture and then was placed in an open jar in a water bath at 37 °C to maintain their body temperature for 25 min and were then returned to the dam. Sham operated control pups were anesthetized for the same average duration as those pups receiving the ligation (~4.5 min). The skin was cut, and a single suture was placed. The carotid artery was not manipulated. Sham operated pups were then placed in the jars and then with the dam for the same intervals as HI littermates. After the 1 h interval with the dam, pups were placed in either hypoxia treatment (8% O_2_:92% N_2_) for 1 h or normoxia for 1 h. After hypoxia or normoxia, pups were placed in normoxia for 4 h and body temperature was maintained at 37 °C in the water bath. At the end of this interval, pups were returned to the dam. 

### 4.4. Neurite Outgrowth Culture and Quantification

On PND10, cerebella were collected from pups. Using a dissection microscope, each cerebellum was separated from the brain stem, and blood vessels and meninges were carefully removed. Each cerebellum was individually placed in cold culture media (KCl, 74.55 g/mole; KH_2_PO_4_, 136.1 g/mole; NaCl, 58.44 g/mole; NaHCO_3_, 84.01 g/mole; Na_2_HPO_4_, 142 g/mole, HEPES free acid, 238 g/mole). Excess media was removed, and cerebella were incubated in trypsin-EDTA followed by the addition of DNase. Tissue was then triturated, and the supernatant was transferred to a 15 mL tube and centrifuged at 4 °C at 800 rpm for 8 min. The supernatant was removed and discarded. The pellet was resuspended in cold neurobasal media supplemented with B27 (Gibco). Cell viability was assessed using trypan blue and a hemocytometer. Cells were then diluted to a concentration of 2 × 10^4^ cells/mL and plated on a poly-L lysine treated glass coverslip [56]. Each coverslip contained cells from a single animal. Half of the coverslips were also incubated with L1-Fc for 2 h at 37 °C in 10% CO_2_. Dishes were then placed in 5% CO_2_ for 48 h. After 48 h in vitro, media was removed and cells were washed with cold phosphate-buffered saline (PBS) three times. Cells were then fixed in 4% paraformaldehyde and washed with PBS three times. Blocking solution was added (3% bovine serum albumin/0.2% Triton X/PBS) for 30 min. Cells were then treated with mouse monoclonal anti-tubulin beta III for 2 h. Finally, cells were treated with the secondary antibody, Alexa 488 anti-mouse IgG for 1 h. Each coverslip was rinsed with PBS and mounted to a slide. Images were captured at 20× magnification using a Zeiss Observer Z1 flourescence inverted microscope and Axiovision software. Images were then quantified with ImageJ software (NIH, Bethesda, MD, USA). Neurite length was measured from the center of the cell soma to the tip of the cell’s longest neurite. Criteria for inclusion were: 1. The neurite was at minimum as long as the soma width 2. The neurite was not touching other neurons or neuronal processes 3. The neuron was not in a cluster of cells. Thirty neurites from each animal were analyzed. 

### 4.5. Histology

Pups were anesthetized with isoflurane and then transcardially perfused with PBS followed by 4% paraformaldehyde. Coronal brain sections were taken on a cryostat at a thickness of 40 μm and mounted to slides. Sections were then dehydrated with a series of ethanol dilutions and stained with cresyl violet. The area of the contralateral and ipsilateral hemispheres at the level of the dorsal hippocampus were measured. The magnitude of tissue loss was quantified as the percentage of tissue in the ipsilateral hemisphere compared to the contralateral hemisphere and was calculated as follows: ipsilateral area/contralateral area × 100. Each sham group consisted of saline (n = 4) and choline treated pups (n = 4) and were combined for analysis. HI groups consisted of 8 pups in each group and each sex. 

### 4.6. Behavior

*Dowel Crossing:* Dowel crossing was conducted on PND30-32. In this task, a wooden dowel was suspended and the rat was placed initially 10 cm from a dark escape box at the ‘target’ end of the dowel. This first trial served as an acclimation trial and the data were not used. On three consecutive days, the rat was again placed on the dowel 10 cm away from the target box. The rat could either successfully cross the distance and enter the box, or not successfully cross the distance and fall onto a padded surface below. A second trial consisted of the rat placed 23 cm from the target box, and the third trial, 36 cm from the target box. Each day the rat could traverse a total of 69 cm. If the rat fell before reaching the target box, the distance traveled was recorded based on even increments marked on the dowel. Data was not used from animals that would not attempt to cross the dowel. The number of animals in each group used for statistical analysis was: Sham, n = 12; HI + Saline, n = 11; HI + Choline, n = 8 for both males and females.

Rotarod: On PND36-39, rats were placed on a rotarod device (Panlab) and acclimated at a constant speed of 10 rpms for 90 s. This was repeated twice. The data from this acclimation day were not used. For three consecutive days, rats were placed on the rotarod and the speed of rotation accelerated for three trials. The device accelerates from 4–40 rpms in 90 s. The amount of time the rat was able to stay on the rod by walking to keep up with the rotation was recorded for each trial. The animals were the same as those tested on the dowel crossing task.

Eyeblink Conditioning: On PND43-44, rats were anesthetized, and a head stage was placed to permit delivery of the unconditioned stimulus (US) and recording of the electromyographic (EMG) activity from the left eye. Head stages were fitted onto the skull with dental cement anchored by screws. Four electrodes (insulated stainless steel wire with a diameter of 0.005 inches) were implanted through the upper eyelid muscle. Two electrodes delivered the US of periorbital stimulation (1 mA, 100 msec), and two recorded EMG activity as a measure of blinks. Pavlovian eyeblink conditioning was initiated between PND50-54. The conditioned stimulus (CS) was a 2.8 kHz tone, at 85 dB, and 380 msec in duration. The CS and US were presented in a trace conditioning stimulus arrangement in which the US was delivered 250 ms after the CS terminated. Conditioned responses (CRs) were EMG signals that were at least 7 ms in duration and 4 standard deviations above a 250 ms baseline period immediately preceding the US. All rats received an acclimation session, in which the animal was attached to the stimulus delivery and data acquisition cable and placed in the conditioning chambers with no stimulus delivery. The following 4 days, 150 trials were presented with an intertrial interval of 30 ± 5 s. Within each block of 10 trials, 1 trial was a CS alone to permit detection of the conditioned response (CR) without contamination by the US. Only CRs expressed during the 230 ms prior to the onset of the US were considered CRs on paired trials, and CRs during, between 400 ms and 730 ms after the offset of the tone on CS alone trials, were considered conditioned responses. Animals that did not exhibit a clear blink, did not exhibit reactivity to the US or exhibited extreme reactivity to the US were not used for statistical analysis. For males, the groups consisted of: Sham + Saline, n = 6; Sham + Choline, n = 7; HI + saline, n = 9; HI + Choline, n = 7. For females the groups consisted of: Sham + Saline, n = 6; Sham + Choline, n = 6; HI + saline, n = 9; HI + Choline, n = 8. Histological analysis of adult animals consisted of a sham group consisting of saline (n = 4) and choline treated pups (n = 4) which were combined for analysis. The male HI + Saline group consisted of n = 11, HI + Choline, n = 8; the female HI + Saline group, n = 11; HI + Choline, n = 8. 

### 4.7. Statistical Analysis

Neurite outgrowth data were analyzed using Kruskall Wallace ANOVA, as these data are not expressed as a continuous variable. Individual groups were compared using the Komolgorov-Smirnov (KS) test to determine presence of group differences. Cerebral volume data were not normally distributed, and therefore analyzed using a Kruskall Wallace ANOVA, with group comparisons made with Dunn’s multiple comparisons tests. Behavioral data were analyzed with Repeated Measures ANOVA with trial block as the within subjects variable and surgery condition (sham or HI) and treatment condition (saline or choline) as the between group variables. 

## 5. Conclusions

Administration of choline from PND1-10 improved several outcome measures including acquisition of the conditioned eyeblink response outcome in male rat pups after HI at term equivalent. In contrast, administration of choline exacerbated the injury from HI in female rat pups and impaired learning related plasticity determined by the conditioned eyeblink response. Our results showing sexual dimorphism in injured brain in response to choline supplementation underscore the importance of using sensitive behavioral measures to determine long-term outcome in male and female brain after neonatal injury.

## Data Availability

The data presented in this study are not publicly available but are available upon request to the corresponding author.
